# Seroprevalence and potential risk factors of contagious bovine pleuropneumonia in the Huambo province of Angola

**DOI:** 10.1038/s41598-026-46690-9

**Published:** 2026-04-03

**Authors:** Antonia Virginia Francisco Pedro, Lucía Manso-Silván, Thibaut Lurier, Maria Luisa Catombela, Armelle Peyraud, André Ravel, Dominique Le Grand, Maria Kialanda Noel, Florence Ayral

**Affiliations:** 1 Faculty of Veterinary Medicine, University José Eduardo dos Santos, Huambo, Angola; 2 Mycoplasmoses Animales, VetAgro Sup, Anses, Lyon University, Marcy L’Etoile, France; 3 ASTRE, CIRAD, INRAE, University of Montpellier, Montpellier, France; 4 Université de Lyon, INRAE, UMR EPIA, VetAgro Sup, Marcy l’Etoile, France; 5 Université Clermont Auvergne, INRAE, UMR EPIA, VetAgro Sup, Saint Genes Champanelle, France; 6 Veterinary Research Institute of Angola, Huambo, Angola; 7 Faculty of Veterinary Medecine, University of Montréal, Saint Hyacinthe, Canada; 8 RS2GP, VetAgro Sup, INRAE, Lyon University, Marcy L’etoile, France

**Keywords:** Angola, cELISA, Contagious bovine pleuropneumonia, Measurement uncertainty, *Mycoplasma mycoides* subspecies *mycoides*, Risk factor, Seroprevalence, Risk factors, Bacterial infection

## Abstract

From June to September 2023, we performed a cross-sectional study to estimate the seroprevalence of contagious bovine pleuropneumonia in the Huambo province of Angola; the study also characterized associated risk factors. We sampled 142 herds belonging to livestock farmers in four municipalities (Caála, Longonjo, Bailundo, and Mungo). A competitive ELISA approach was used to detect the presence of antibodies against *Mycoplasma mycoides* subspecies *mycoides* (*Mmm*) in 1647 cattle. Herd-level seropositivity was determined using a threshold that accounted for test measurement uncertainty in the laboratory. Data on herd characteristics were collected from livestock farmers using a questionnaire. We explored the relationship between 14 potential risk factors and herd seropositivity using logistic regression models. Overall, apparent herd-level seroprevalence was 55.6% (95% CI 46.9–63.1%). The likelihood that a herd was seropositive was related to the area where cattle had been purchased; it was significantly higher when animals came from the southern region of Huambo (odds ratio: 5.5; 95% CI 1.4–17.5). Although our study was limited by its sampling design (we lacked comprehensive information on the province’s farms), our findings suggest that *Mmm* infections may occur at significant levels in Huambo and highlight distribution patterns that could guide disease control strategies in livestock farming systems.

**Supplementary Information:**

The online version contains supplementary material available at 10.1038/s41598-026-46690-9.

## Introduction

Contagious bovine pleuropneumonia (CBPP) is a severe respiratory disease in cattle caused by *Mycoplasma mycoides* subspecies *mycoides* (*Mmm*)^1^. CBPP is listed by the World Organization for Animal Health (WOAH) as a notifiable diseases and is among the six diseases subject to official recognition of disease-free status. It is one of the main causes of disease-related cattle mortality in Africa^2^. Beyond animal health impacts, CBPP has important socioeconomic consequences. The disease can reduce livestock productivity and threaten the livelihoods of smallholder farmers. Outbreaks may also lead to economic losses due to trade restrictions and control measures. In many rural areas, cattle provide food, income, and draft power; therefore, the disease can significantly affect food security and household resilience^3^. Despite control efforts, CBPP remains endemic in most African countries and continues to threaten livestock production.

CBPP mainly affects domestic cattle (*Bos taurus* and *Bos indicus*), and no wildlife reservoir has been identified. The disease spreads primarily through aerosols from bronchial secretions of infected animals, requiring repeated close contact^4,5^. The transmission risk increases at shared grazing areas, watering points, and during seasonal movements such as transhumance, particularly in the dry season^6,7^. Indirect transmission through contaminated surfaces has not been reported^4^.

*Mmm*’s incubation period ranges from 3 weeks to 6 months, averaging 4 to 5 weeks^8^. Classical, acute CBPP is characterized by severe serofibrinous pleuropneumonia, usually affecting one lung, pleurisy, edema of the interlobular septa, and hepatization of lung tissue. Chronically infected animals may develop encapsulated lesions known as sequestra. The *Mmm* can persist for over two years in subclinical or healthy carriers, contributing to long-term disease persistence^4^. Infection is confirmed by isolating and identifying the bacteria and by using PCR and serological tests^9^. To serologically diagnose CBPP at the herd level, the WOAH recommends using either the competitive enzyme-linked immunosorbent assay (cELISA) or the complement fixation test (CFT). The former is commonly used in seroprevalence surveys in Africa^10^.

CBPP control and eradication rely on several strategies, including vaccination, isolation, and slaughter. The disease remains endemic in many eastern, central, western, and southern African countries because control efforts have been constrained by several factors, including declines in veterinary services, an absence of financial resources, and a lack of livestock traceability^11^. In addition, there are gaps in epidemiological knowledge: the proportion of infected animals that develop disease, within-herd prevalence, and the factors driving herd infection (e.g., pastoralism, animal purchases, season) are either unknown or subject to debate, depending on the studies^12–16^. These gaps limit efforts to develop effective control and prevention strategies.

Although CBPP spread throughout Angola during the 27 year civil war (1975–2002), the disease was eliminated from the central and northern provinces via depopulation. However, the government was constrained in its ability to comply with health and safety regulations related to animal trade and transport (Angola Presidential Decree no. 4 of August 13, 2004—Animal Health Law), which caused a resurgence in cases in these provinces^17^. Starting in 1921, animals were vaccinated every year through vaccination campaigns^17^. In 2014, an economic recession interrupted these efforts. In 2016, at the regional network meeting for harmonizing the CBPP eradication strategy in Southern African Development Community (SADC) countries, it was agreed that prophylactic measures would be implemented to eradicate the disease by 2030^17^.

In Angola, the southern provinces of Namibe, Cunene, and Lubango have been reported to be affected^17^. Namibe has been the only province to be serologically surveyed for CBPP with a high apparent herd-level seroprevalence (94.3%)^18^. Additional information from slaughterhouse inspections conducted by veterinary services revealed the disease through the observation of macroscopic lesions consistent with CBPP in 43.8% (7/16) of the selected cattle^19^. However, these data do not provide clarity on regional prevalence patterns. Southern Angola is also strategically important for disease control due to its border with Namibia, where cases of CBPP have been regularly reported^20^.

The cattle sector in Angola includes both commercial and traditional farming systems, which differ in production objectives, management practices, and use of external inputs such as veterinary services and mechanization. These differences influence herd management and patterns of animal contact, potentially affecting disease transmission dynamics^21^. Traditional farming dominates the agricultural sector and plays a key role in rural livelihoods and food security. Approximately 2.22 million families are involved in this sector, accounting for around 90% of the country’s agricultural production^22^. Cattle production is largely based on traditional systems, with about three million cattle raised on family farms compared with approximately 260,000 on commercial farms^23^.

The goal of this study was to assess apparent herd-level CBPP seroprevalence and the associated risk factors in the Huambo province of Angola with a view to informing the control strategies used by authorities and traditional livestock farmers. We focused on traditional farms, which account for 75% of cattle production in Angola. We examined the potential for a south–north gradient of *Mmm* infections, given that the Huambo province is located just north of an area where CBPP seroprevalence is known to be high. The two northernmost municipalities, Bailundo and Mungo, and the two southernmost municipalities, Longonjo and Caála, were considered in the study.

## Results

### Cattle herds

Blood samples were collected from 1647 cattle (839 females and 808 males). Herd size varied from 4 to 22 cattle (median = 10 cattle).

### Seroprevalence

Overall, 79 of the 142 herds were seropositive, corresponding to an apparent herd-level seroprevalence of 55.6% (95% CI [47.0–63.8%]).

**Fig. 1:**
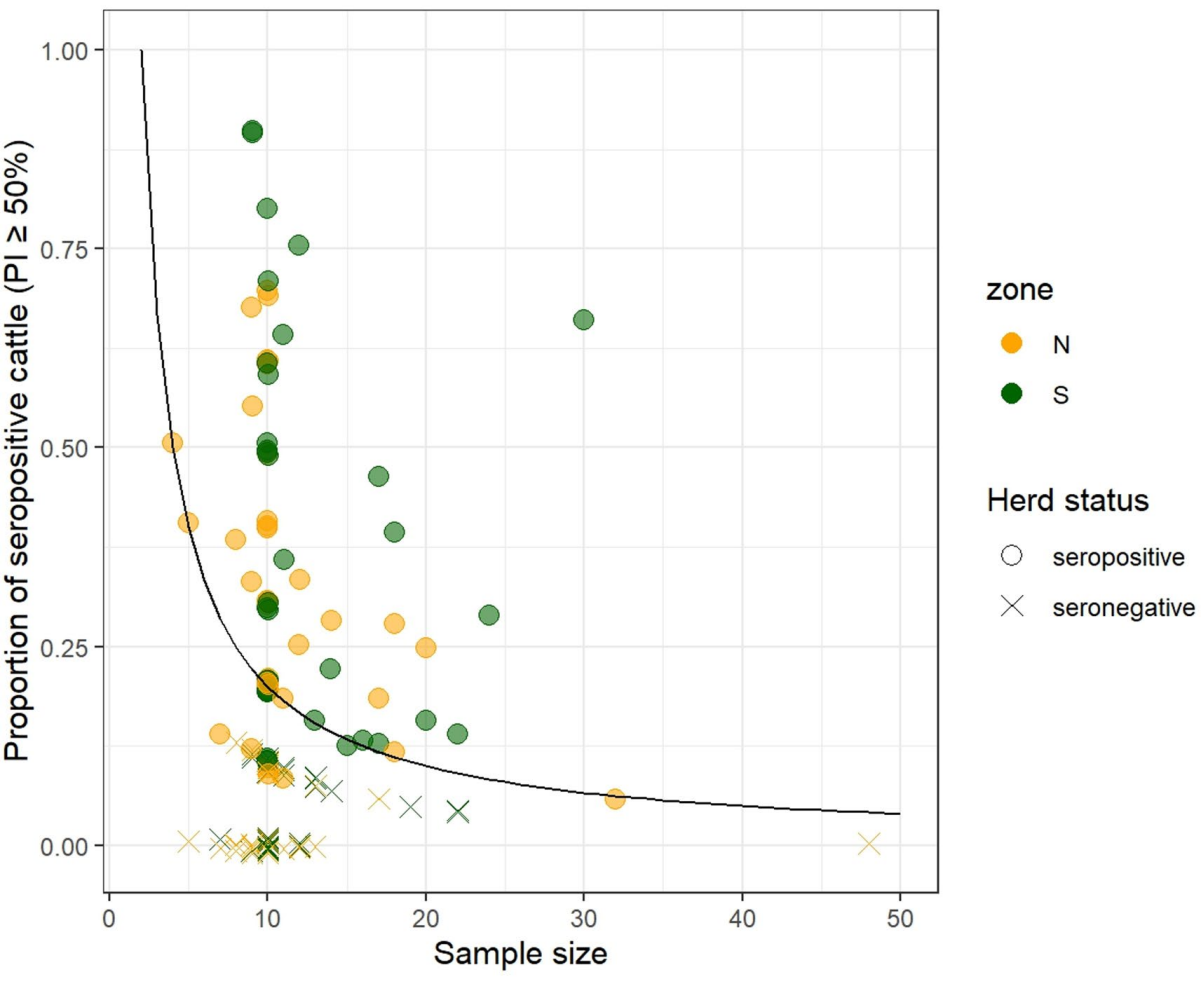
Relationship between the number of cattle sampled (a proxy for herd size) and within-herd seroprevalence in the southern (green) and northern (yellow) regions of the Huambo province. A herd was classified as seropositive (circle) if at least one animal had a PI value greater than or equal to 62.5 or at least two animals had PI values between 50 and 62.5; otherwise, the herd was classified as seronegative (cross). Seropositive herds above the curve had at least 2 seropositive animals with PI values greater than or equal to 50.

The relationship between herd size and within-herd seroprevalence across the two regions suggests that herds in the southern region had higher seroprevalence levels (Fig. 1).

**Fig. 2:**
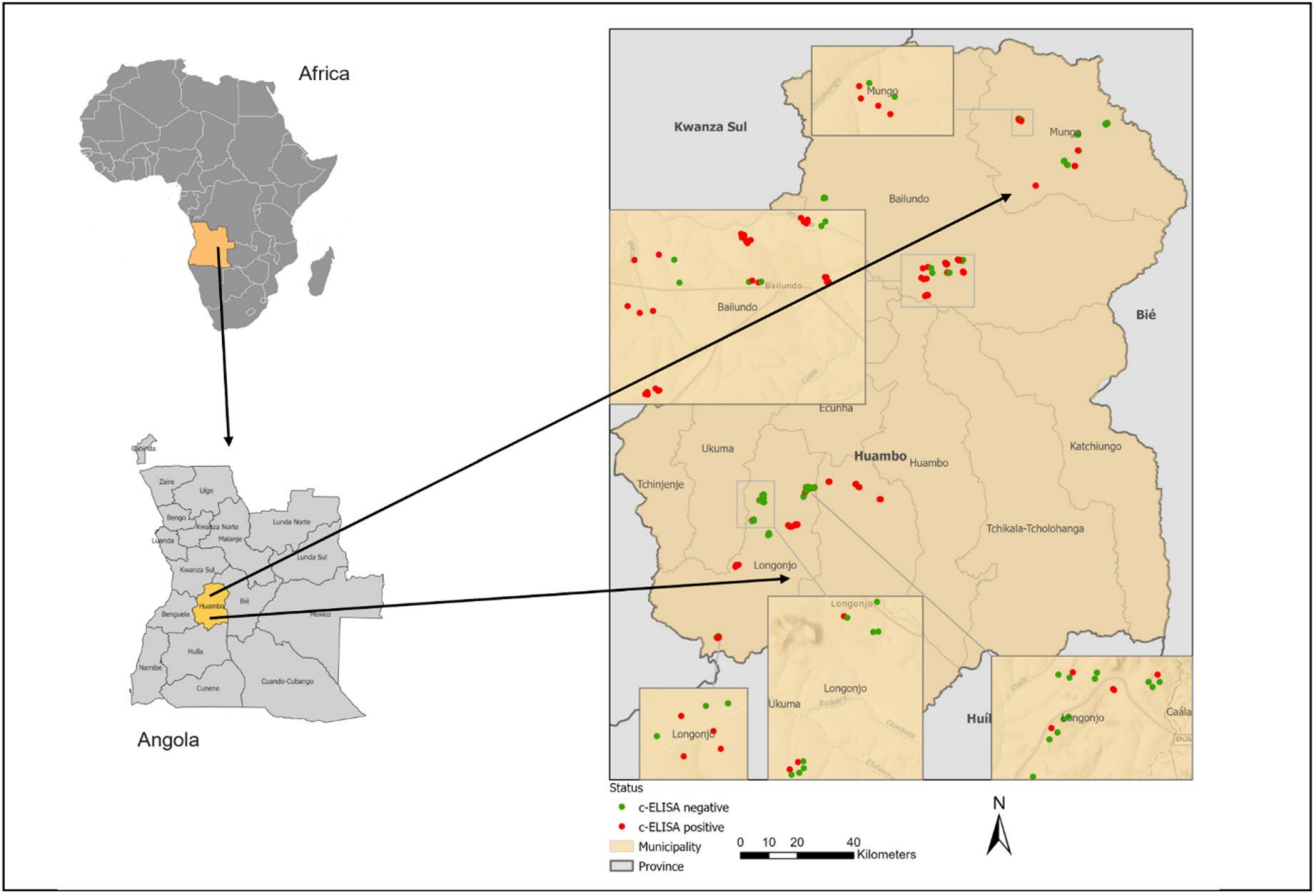
Spatial distribution of seropositive and seronegative herds sampled in this study in the Huambo province in 2023 (QGIS Development Team. QGIS Geographic Information System, version 3.1, Open Source Geospatial Foundation Project [2024]).

The spatial distribution of the seropositive and seronegative herds is shown in Fig. 2.

### Relationship between the variables and CBPP seropositivity

Considering the spatial distribution of the tested negative and positive herds in North and South, there was no significant difference between the two regions (Table 1).

**Table 1:** Apparent herd-level CBPP seroprevalence in 2023 in the Huambo province.

Region	No. herds	No. seropositive herds	Seroprevalence (95% CI)
Northern	75	43	57.0% (45.3%–68.5%)
Southern	67	36	53.0% (41.1%–65.8%)
Total	142	79	55.6% (47.0%–63.8%)

The following variables were statistically associated with herd seropositivity (*p* ≤ 0.20): mixed herd, presence of vaccinated animals, purchase area, antibiotics usage, and most recent animal purchase (Supplementary Table S1). The full multivariable model included these five variables. Among the six models with the lowest AIC values, four of them have a ΔAIC < 2. The most comprehensive model was retained as the AIC value was in a meaningful ΔAIC ≤ 2 range and it was the most informative. The odds ratios varied only marginally across the top three models (Supplementary Table S2).

The model’s coefficients were used to calculate the odds ratios for each of the variable values (Table 2). Notably, the likelihood of a herd being seropositive was 5.5 times higher when cattle had been purchased in the southern region (OR = 5.5; 95% CI [1.5–21.3]), regardless of whether the herd was located in the northern or southern region. This likelihood was 4.5 times higher when the most recent animal purchase had taken place more than 6 months ago (OR = 4.5; 95% CI [1.5–14.9]).

**Table 2:** Odds ratios from the final model of the relationship between the animal husbandry variables and herd-level CBPP seropositivity.

Variable value	OR (95% CI)	*p*-value
Mixed herd (yes)	2.7 [0.8–11.1]	0.123
Most recent animal purchases > 6 months	4.5 [1.5–14.9]	0.010*
Presence of vaccinated animals (all)	3.4 [1.0–14.7]	0.065
Purchase area: province south of Huambo	1.3 [0.1–13.2]	0.832
Purchase area: other province & northern Huambo	1.25 [0.05–17.60]	0.870
Purchase area: other province & southern Huambo	3.8 [0.7–27.3]	0.168
Purchase area: southern Huambo	5.6 [1.6–21.3]	0.010*
Purchase area: northern & southern Huambo	1.8 [0.4–8.6]	0.424
Antibiotics usage (yes)	0.4 [0.1–1.1]	0.077

*variable associated with a p-value below 0.05.

## Discussion

### Optimizing detection of seropositive herds

We used competition ELISA (cELISA) instead of the complement fixation test (CFT) because cELISA can better detect chronic forms of CBPP^24^. In areas where the disease is endemic, infection spreads slowly through herds, and not all animals are infected at the same time^8^. Because the presence of IgM antibodies is temporary, CFT has a limited ability to detect chronic CBPP. In contrast, cELISA can detect the presence of IgG antibodies, which persist for several months^25^. We thus chose to use cELISAs to identify seropositive animals given that we were not sampling during an outbreak.

In the context of CBPP serological testing, cELISA has a specificity level of 99%, while CFT has a specificity level of 98%; both have a sensitivity level of around 60%^26^. To compensate for this lower degree of sensitivity, the following is recommended: (1) cELISA results should be interpreted at the herd level, (2) sampling should focus on animals with a history of respiratory disease, and (3) within-herd sample sizes should be large enough to assess herd serostatus with a high degree of statistical confidence. In this study, we did not have access to reliable epidemiological data or information about earlier cases of respiratory disease resembling CBPP in the herds sampled. We therefore attempted to boost sensitivity by sampling adult animals, which are more likely to have been infected^27,28^.

We followed the recommendations of the Regional Sahel Pastoralism Support Project (PRAPS, *Projet Régional d’Appui au Pastoralisme au Sahel*), which receives World Bank funding^29^ to optimize the detection of seropositive herds. PRAPS recommends assessing herd serostatus in a way that accounts for test measurement uncertainty (see Methods section “Serological testing and interpretation”). We found that apparent herd-level seroprevalence was 65% (95% CI [56.8–72.3%]) when using the manufacturer’s threshold and 31% (95% CI [23.5–39.2%]) when accounting for cELISA uncertainty. PRAPS suggests that using the manufacturer’s threshold is likely to overestimate herd-level seroprevalence, while using an approach that accounts for cELISA uncertainty is likely to underestimate herd-level seroprevalence. Thus, it is important to account for uncertainty and strike the right compromise when interpreting cELISA results to improve seroprevalence estimates. More reliable seroprevalence estimates allow more effective management measures to be taken. PRAPS states that a herd should be classified as seropositive if at least one animal has a percentage of inhibition (PI) value that is greater than or equal to the kit positivity threshold plus measurement uncertainty (PI ≥ 62.5) or if at least two animals have PI values between the kit positivity threshold and the kit positivity threshold plus measurement uncertainty (62.5 > PI > 50). Otherwise, the herd should be classified as seronegative. We applied this threshold while also carefully determining the appropriate within-herd sample size, which allowed us to optimize diagnostic sensitivity when determining herd-level serostatus.

### Seroprevalence

Based on the 142 herds, apparent herd-level seroprevalence was 55.6% (*i.e.*, percentage of seropositive herds based on the above PRAPS threshold); this sample size yielded a satisfactory level of precision (SD ± 0.2). In this study, seropositivity reflected the presence of specific post-infection antibodies. We assumed there was no influence of vaccination-elicited antibodies because sampling was carried out at least 12 months after the last vaccination campaign. Some herds had not been vaccinated for over 24 months (n = 26) while others had never been vaccinated at all (n = 14). We thought it was unlikely that the herds contained recently vaccinated animals that had arrived from other countries where vaccine campaigns had occurred more recently (*i.e.*, in the three months preceding our sampling efforts). Although the within-herd sample size was calculated assuming a theoretical sensitivity of approximately 99%, this limitation is unlikely to have underestimate the apparent herd-level seroprevalence, as the herds were small and all animals in each herd were tested. As we followed PRAPS recommendations, we attained a high level of specificity, allowing us to identify seropositive herds. At the same time, we might have underestimated herd-level CBPP seroprevalence because of the lack of sensitivity, as noted above.

Our estimate of apparent herd-level seroprevalence in the Huambo province of Angola is higher than the estimate of apparent herd-level seroprevalence reported for Nigeria (27.2%, 95% CI [19.6–35.9%]^30^. The latter study was able to implement random sampling, which limited some sources of bias (*e.g.*, the selection bias of herds from villages with a greater interest in CBPP). Another study in 2004 in Ethiopia observed an apparent herd-level seroprevalence of 30.4% (95% CI [19.1–44.2%], n = 56)^31^, even though the researchers utilized an approach with greater sensitivity than ours. Notably, they focused on animals associated with clinical episodes of disease, and a herd was classified as seropositive if at least one animal displayed a PI value greater than 50 during cELISA testing, irrespective of measurement uncertainty. This lower level of apparent seroprevalence in Ethiopia could be explained by the country’s epidemiological situation, which may have been shaped by 30 years of vaccination campaigns^32^. In contrast, in Angola, vaccination campaigns against CBPP have been inconsistent. They only happen when the government has adequate financial resources, and often a select number of regions are prioritized. For example, we encountered herds that had not been vaccinated in the last two years, if ever. CBPP seroprevalence tends to be higher in herds in extensive farming systems that experience inconsistent vaccination^33^.

Our estimate of apparent herd-level seroprevalence was lower than that observed in Mali (85.9%; CI 95% [80.4–90.1%]) during a study that conducted routine sampling (n = 8,007 cattle from 199 herds)^16^. Another study conducted in the Namibe province of Angola estimated that apparent herd-level seroprevalence was 94.3% (CI 95% [79.4–99.0%]; n = 1226 cattle from 35 herds)^18^. Neither of these studies followed PRAPS recommendations, and their subsequent failure to account for measurement uncertainty^29^ might have contributed to their higher seroprevalence estimates. Indeed, both studies classified herds as seropositive if they contained at least one animal with a PI value of 50 or higher, which could have led to overestimates of herd-level seroprevalence. In addition, this difference in seroprevalence estimates could be explained by herd size. In the two above studies, researchers sampled cattle herds that averaged 40 animals in size; in contrast, in our study, mean herd size was 12 animals. Three previous studies found that herd size was a potential risk factor, herds containing larger numbers of animals were more likely to be seropositive for CBPP^30,34,35^. The hypothesis is that the effective contact rate between CBPP-infected cattle and susceptible cattle is higher in larger herds^36^.

### Potential risk factors related to CBPP herd serostatus

Our results raise the question as to whether herd composition is significantly associated with CBPP seroprevalence. Notably, it seems more likely that CBPP would have a greater likelihood of appearing in mixed herds, meaning herds that contain cattle and small ruminants. A study in Nigeria found that mixed herd composition was a significant risk factor for CBPP seroprevalence (univariate analysis: *p*-value < 0.001)^13^. *Mmm* is specific to cattle, but *Mmm* isolates have been obtained from mixed herds^37^. Although small ruminants do not appear to play an important role in CBPP epidemiology, it is possible that they serve as a reservoir in the disease’s transmission to cattle. This question could be explored in future research.

In addition, we found that herds were significantly more likely to be seropositive if they contained animals that had been purchased in the southern region (Table 2). The herd distribution across purchase areas was unbalanced, with smaller sample sizes in some categories (e.g., 3–12 herds) (ST1), largely reflecting that most farmers source cattle from the south, resulting in limited observations for northern purchases. This imbalance likely contributes to the wide confidence interval for these categories and reduced precision of the estimate. Although further data is needed to confirm these results, this observation is consistent with the risk level associated with the lack of biosecurity measures and reported occurrences in the area. The southern region of Huambo hosts the province’s largest animal market^38^, which is unregulated. Sellers do not need to furnish animal health certificates, and the animals’ origins are unknown. Cattle are simply bought by livestock farmers and then introduced into existing herds. According to the Angola Ministry of Agriculture, animal densities and levels of pastoralism are higher in the southern region than in the northern region. Furthermore, the southern region is adjacent to the Angolan provinces with ties to Namibia, a country where CBPP outbreaks have been reported^20^ and which develops trade with the southern provinces of Angola; the trade being responsible for maintaining and spreading the disease within countries and among neighboring countries^4^.

We also found that a herd was significantly more likely to be seropositive if the most recent animal purchase occurred longer than 6 months ago (OR 4.5, 95% CI [1.5–14.9] (Table 2), which seems counterintuitive if animal purchases are a risk factor. It should be noted that the number of livestock farmers who had purchased an animal within the last 6 months was relatively small (n = 28 herds). This result may have been influenced by our sampling approach or by farmer behavior, such as the decision to avoid animal purchases after episodes of any disease resembling CBPP. In addition, the etiological agent of CBPP has a prolonged incubation period (*i.e.,* 1 to 6 months)^8^, and animals may be slow to develop a serological response. After 6 months, antibodies may still be detectable, especially since cELISA testing can detect antibodies during the chronic phase of the disease^25^. It is also possible that there were previous outbreaks that went unreported or undiagnosed to which animals may have been exposed. Furthermore, some farmers buy their animals at informal markets, which are one of the main sources of the disease because there are no health control measures^39^. There is a risk that cattle at informal markets come from areas where CBPP prevalence is higher, such as southern Angola. There is also the risk of animals being introduced into the herd without undergoing quarantine, a common practice among farmers. Thus, in summary, the counterintuitive result we obtained illustrates the limitations of cross-sectional studies: it is impossible to know the temporal relationship, let alone the causal relationship, between two associated variables.

Overall, 91 livestock farmers indicated that they used antibiotics (herd seropositivity = 48%), and 51 livestock farmers indicated that they did not (herd seropositivity = 68%). Antibiotics are not recommended in the case of CBPP because they can lead to the development of pulmonary sequestra and chronic carriage, thus creating potential sources of infection^7^. Yet, farmers commonly use antibiotics^7^, especially farmers who make treatment decisions based on personal judgment^40^.

### Potential risk factors unrelated to CBPP herd serostatus

We examined other potential factors that could affect transmission, including the use of communal pens, contact with other herds during watering or grazing, and whether animals were quarantined before introduction into the herd. None were found to influence herd seropositivity. Our results do not support the hypotheses that herd-level CBPP occurrence and spatial distribution patterns are driven by transhumance, the mixing of cattle in pastures or at watering points, or the introduction of new animals into herds^7,13,18^. It could be that we did not observe a statistically significant pattern because of methodological factors, such as sample sizes that were not large enough to capture weaker effects (OR < 5).

Modeling results from previous research indicate that a herd’s probability of becoming infected is positively related to that herd’s contact rate with other herds^7^.The livestock farmers who participated in our study did not practice transhumance, which would have resulted in more contact among herds^41^. The only place where different herds came together was in pastures and around watering points, and these interactions were limited to herds from the same village. As a result, contact intensity and frequency were lower than they would have been in the context of transhumance, which might have resulted in the lower levels of seropositivity seen in our study. Animal husbandry practices remain the main factors that determine contact rates among herds^13^. In addition, there may have been some confusion in the farmers’ responses to the question about whether their herds grazed in pastures shared with other herds because the answer was a simple yes or no. Indeed, pastures could be shared according to a staggered grazing schedule (several herds using the same pasture at different times), which would have meant that the herds did not come in contact with each other. Thus, a “yes” might or might not have indicated contact among herds.

### Study limitations

Herds within the same village may share similar serostatus because of close contact. In the study, villages contained 2–10 herds, and herd serostatus varied widely. Among 42 villages, 12 were entirely seronegative, while 30 had at least one seropositive herd. In 19 of these villages, herd seropositivity exceeded the mean apparent prevalence of 55.6%, and 30% of villages accounted for 70% of seropositive herds. Thus, sampling several herds within seropositive villages may have introduced selection bias, potentially overestimating herd-level prevalence. However, herds were managed in a self-contained way without pastoralism or shared watering points, which have limited a potential village effect.

In addition, its effect on the odds ratio (OR) appeared negligible. Indeed, when we ran a mixed model incorporating village as a random variable, the OR remained approximately the same (data not shown). Ideally, we would have conducted two-stage sampling (first the village, then the herd) if we had had a list of existing herds prior to beginning the study; we would then have been able to assess the village effect through mixed-effects modeling.

Among the six models with the lowest AIC values, four had a ΔAIC < 2, indicating that they were essentially equivalent in terms of goodness of fit, we retained the most comprehensive model including the variables mixed herd, follows vaccination schedules, purchase area, most recent animal purchase and antibiotic use, which was considered the most biologically meaningful according to the literature. Importantly, the odds ratios estimated in this four-variable model were of the same order of magnitude as those obtained in the other top-performing models, suggesting that the main associations identified are robust to model specification (ST2). This consistency supports the relevance of the selected model for interpretation.

### Recommendations

Our results show that CBPP is present in the Huambo province of Angola. These data provide a snapshot of CBPP seroprevalence in the province, which can be used as a starting point from which to broaden our knowledge about the disease. Greater understanding of CBPP seroprevalence and distribution patterns across the study regions can help us develop recommendations for limiting the disease’s spread and designing eradication strategies.

Current CBPP control measures include certifying herd seronegativity, an approach that is not realistic in Angola given the lack of cattle traceability. In addition, it might be useless, since seroconversion is short-lived and asymptomatic carriers cannot be detected. Vaccination remains the most feasible option. While it is impossible to vaccinate all herds for economic reasons, vaccination campaigns could selectively target certain herds. Notably, we found that purchasing animals in the southern region of the province and seropositivity was statistically linked, which suggests that this region could become the focus of vaccination campaigns. The southern region is also home to the province’s largest livestock market. Therefore, individuals selling cattle at this market should be encouraged to vaccinate their animals.

Future research could take the form of a preliminary study to characterize seroprevalence among cattle herds whose members are sold at the market. The results could indicate whether prioritizing the vaccination of this population would be worthwhile. It could also be useful to create a list of existing herds and their locations because preliminary survey efforts could then randomly select herds for sampling and thus limit bias.

Implementing these recommendations will require the availability of serological tests, the presence of adequate infrastructure, the accessibility of consumables, and a certain number of skilled personnel to carry out the tests. During this study, analyses were performed in local laboratories run by the Angola Ministry of Agriculture, showing that the country should be able to routinely conduct serological testing as part of efforts to fight CBPP.

## Conclusion

Our results show that cattle herds in the Huambo province of Angola were frequently exposed to *Mmm,* the pathogen that causes CBPP. Our main finding was the relationship between the herd CBPP serostatus and the cattle purchase area. In addition, a herd was more likely to be seropositive when it contained animals from the southern region that had been acquired more than 6 months previously. A disease control plan should be implemented by government officials to reduce the economic consequences associated with the high level of apparent seroprevalence that we observed. Disease control efforts should identify strategic areas to target, such as markets, and focus on related herds during vaccination campaigns. It is also crucial to increase the availability of serological tests to conduct regular monitoring.

## Materials and methods

### Study areas

**Fig. 3:**
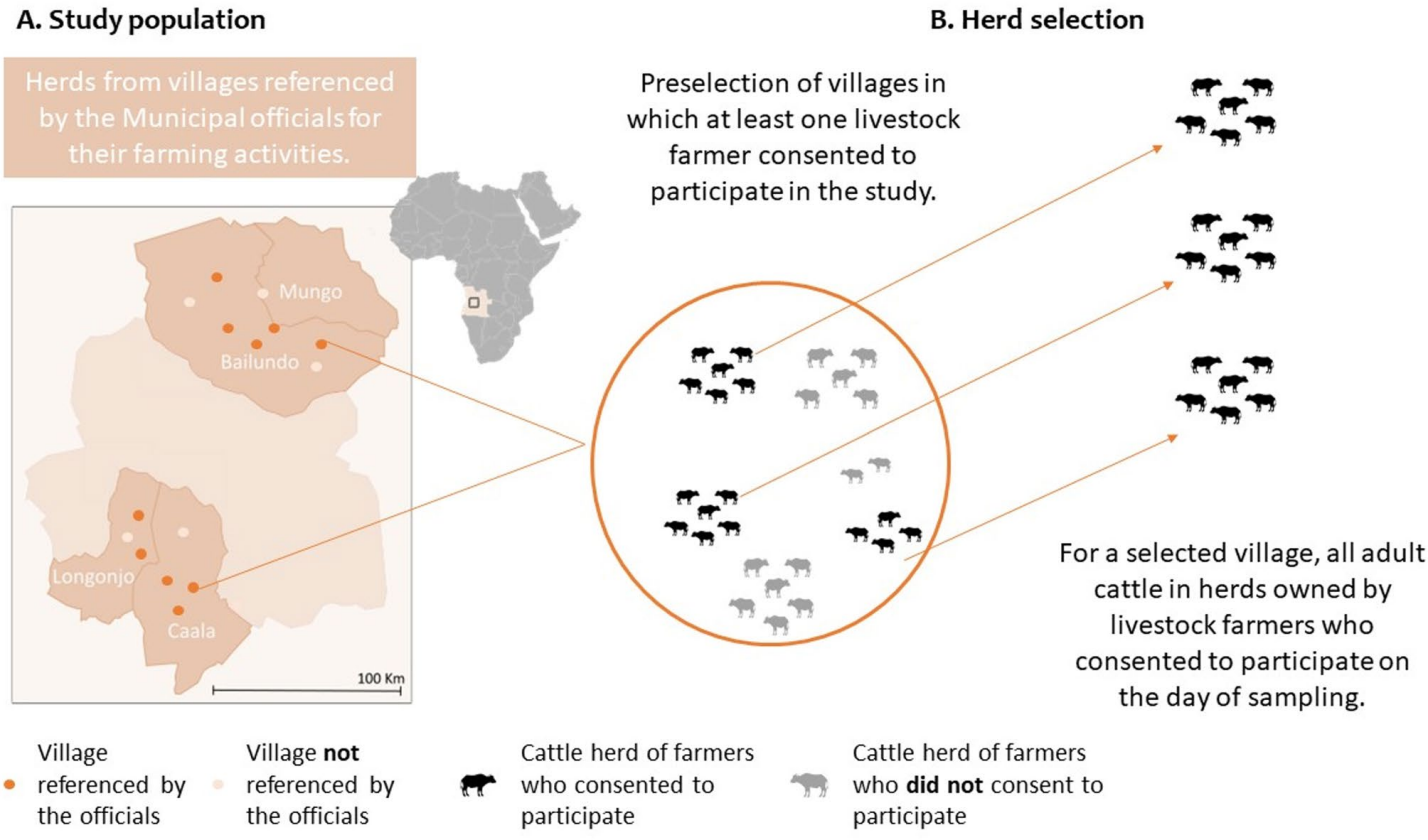
(**A**) The study population was the cattle herds on traditional farms in villages that municipal officials had recorded as having cattle herds; the livestock farmer owning the herd also had to have consented to participate. (**B**) The herds selected were the herds owned by livestock farmers who consented to participate (Maps done with Inkscape Project 2023, version 1.3 [0e150ed6c4, 2023-07-21]).

Administratively, Angola is organized into provinces, municipalities, districts, and villages. Huambo is one of the country’s 21 provinces. It is located in southern central Angola (-12°30′0.00" S 15°40′0.12" E) and is divided into 17 municipalities. The Huambo province extends over approximately 35,771 km^2^, which is around 3% of the country’s total surface area. From north to south, Huambo is a maximum of 260 km long; from east to west, it is 180 km at its widest point^38^. Huambo ranks second nationally in terms of its number of cattle^42^. The study was carried out in the municipalities of Longonjo and Caála (southern region of Huambo) and in the municipalities of Bailundo and Mungo (northern region of Huambo) (Fig. 3A).

Huambo has two distinct seasons: the dry season, which lasts from May to August, and the rainy season, which lasts from September to April^23^. About 95% of rainfall occurs during these latter months, with peaks in December and March. The mean temperature ranges from 18 to 23 °C throughout the year. The mean daily maximum temperature ranges between 25 and 27 °C, and the mean minimum temperature ranges between 11 and 13 °C. September and October are the hottest months, and June and July are the coolest. The heaviest rainfall occurs during the warmer summer months, particularly between October and April. Regional features such as land use, climate, and topography vary between northern and southern Huambo.

### Study population

A cross-sectional study was carried out to estimate CBPP seroprevalence at the herd level and to identify potential risk factors influencing herd serostatus. Given the latter objective, the herd was the epidemiological unit. A herd was defined as a group of cattle that was independent of other groups of cattle and that belonged to a single owner. Our study population was herds of cattle reared within traditional farming systems.

According to a report on family farms/fisheries and aquatic farms, the Huambo province contains a total of 191,423 cattle, which mainly occur on traditional family farms^42^.

To optimize the detection of seropositive herds, the Regional Sahel Pastoralism Support Project (PRAPS; *Projet Régional d’Appui au Pastoralisme au Sahel*^29^), recommends including cattle with a history of respiratory disease. To limit any potential bias related to farmer memory, we focused on adult cattle (> 12 months old) because animals between one and three years old are more likely than calves to be seropositive^41^. The animals were selected independently of sex because previous research has shown that sex is not a risk factor^43^.

### Sampling design

Officials from each municipality (Longonjo, Caála, Bailundo, and Mungo) collaborated with traditional regional chiefs to furnish a list of local livestock farmers. These farmers were contacted in advance to ask if they wished to participate in the study; all who agreed were included (Fig. 3B).

We used the EpiTools application^44^ to determine the number of herds we needed to sample in the northern and southern regions. Specifically, we wanted to sample enough herds to detect a significant regional difference if such a difference were present. When performing the calculation, the expected odds ratio (OR) was 5, the expected percentage of animals exposed within the controls was 5%, the confidence level was 95%, and the desired power was 80%. We found that we needed to sample at least 67 herds in each region (n = 134 in total).

We determined the number of animals that needed to be sampled based on the formula developed by Cameron and Baldock (1998). This formula accounts for the probability of detecting diseased animals, imperfect testing, and finite population size^45^. We used an expected within-herd seroprevalence of 13%^46^ and a precision level of 5%^47^; we assumed imperfect sensitivity and a cELISA specificity of 0.99 for both. We determined the minimum number of animals we would need to test to identify the presence of a single seropositive animal in a herd (although the herd would still be classified as seronegative in such a case). The results indicated that, for a herd of 30 adults, it was necessary to sample 28 individuals. For herds containing fewer than 30 adults, we sampled all individuals.

### Blood and serum samples

Between June and September 2023, we sampled 142 herds in 42 villages. There were 21 villages and 67 herds in the southern region, and 21 villages and 75 herds in the northern region. After an animal had been properly restrained, 5 ml of blood was drawn from the jugular vein using a vacutainer tube without anticoagulant to promote clot formation. The sample was then stored in a box kept at 4 °C (± 2 °C) in the field. Once all the samples had been collected for the day, they were transported to the Huambo Regional Veterinary Laboratory. In the laboratory, the samples were centrifuged at ~ 800 g for 5 min. The sera were collected in Eppendorf tubes and stored at −20 °C until further analysis. Each serum sample was clearly labeled with identifying information and a tag indicating that it needed to remain at subzero temperatures.

None of the animals had been recently vaccinated (< 3 months ago), which is important to prevent any cross-reactivity with vaccination-elicited antibodies (T1/44 strain) during cELISA testing^48^.

### Approval, permission, and informed consent

Local government authorities and the livestock farmers who owned the cattle consented to the animal sampling procedure ahead of time and again at the time of sample collection. Consent was obtained in person from the livestock farmers: we explained the study’s objectives and requested access to the cattle. At the time of the interview (see below), participants signed an informed consent form. They were informed of the following: (1) their participation was voluntary, (2) their responses would be kept confidential, and (3) they could withdraw from the study at any time without any risk of reprisal. Following the publication of this article, we and the municipal authorities will share the study’s results with the livestock farmers. The entire process was carried out in accordance with relevant guidelines and regulations (French decree no. 2018-687 and Regulation [EU] 2016/679 of the European Parliament and of the Council of 27 April 2016 on the protection of natural persons with regard to the processing of personal data and on the free movement of such data [GDPR]).

### Animal ethics statement

Our fieldwork in Angola was reviewed and approved by an ethics committee from VetAgro Sup (Agreement no. 2231 obtained on 2022/04/07). All procedures for animal sampling complied with ARRIVE guidelines and the ethical standards of the relevant French and European regulations on the care and use of animals (French decree of 2007/04/06 and Directive 2010/63/EC).

### Serological testing and interpretation

We carried out cELISAs using a CBPP cELISA Kit (P05410/10, CIRAD/IDEXX, Montpellier, France) in accordance with the manufacturer’s instructions. Optical densities (ODs) were measured at 450 nm using a spectrophotometer (SpectraFluor, Tecan, Crailsheim, Germany).

We estimated the degree of measurement uncertainty (95% confidence interval [CI]) around the kit’s positivity threshold in the laboratory at the Institute of Veterinary Research of Angola (IIVA). For this purpose, we employed 18 weakly positive control samples (C +) from the kit, and we considered all the variables likely to have an impact on the results (*i.e.,* technician, materials used, day). Measurement uncertainty was defined as twice the standard deviation of the individual C + values expressed in percent inhibition (PI): δi = 6.28 × 2 = 12.5 PI.

The positivity threshold indicated in the kit was 50 PI. We classified a herd as seropositive if at least one animal in the herd had a PI value greater than or equal to the kit positivity threshold plus measurement uncertainty (PI ≥ 50 + 12.5, or PI ≥ 62.5) or if at least two animals in the herd had a PI value between the kit positivity threshold and the kit positivity threshold plus measurement uncertainty (62.5 > PI > 50). We adopted this approach based on PRAPS project recommendations.

Apparent herd-level seroprevalence (%) was calculated by dividing the number of seropositive herds by the total number of herds tested and then multiplying the result by 100. The within-herd seroprevalence was calculated by dividing the number of seropositive individuals by the total number of tested individuals in the herd. The within-herd seroprevalence was used to describe the potential variation of seropositivity in north and south municipalities. The 95% CIs for seroprevalence were estimated using the binomial exact method (R v. 4.2.0).

### Questionnaire design and use

The practices addressed in the questionnaire were selected based on previous research^13^. The questionnaire responses provided information about 20 variables, of which 6 were found to be redundant. Thus, 14 variables were employed in our analyses (Table 3).

**Table 3:** Variables related to animal husbandry practices addressed in a questionnaire administered to livestock farmers to identify potential CBPP risk factors for herds in the Huambo province in 2023.

Variable	Definition	Variable values
Herd region	Geographical region where herd is located	northern
		southern
Mixed herd	Presence of both cattle and small ruminants in herd	no
		yes
CBPP knowledge	Level of livestock farmer or owner knowledge about CBPP	none
		poor^1^
		good^2^
		very good^3^
Prior disease in herd	Livestock farmer/owner reported previous CBPP-like disease in herd	no
		yes
Follows vaccination schedule	Participation in government-run vaccination campaigns	no
		yes
Presence of vaccinated animals	At least some animals in herd were previously vaccinated against CBPP	adults only
		all animals
Purchase area	Area where animals were purchased	northern Huambo
		province south of Huambo
		province other than Huambo & northern Huambo
		province other than Huambo & southern Huambo
		southern Huambo
		northern & southern Huambo
Purchase frequency	Annual frequency of animal purchases	once per year
		twice per year
		three times per year
Most recent animal purchase	Time period over which last animal purchase occurred	< 6 months
		> 6 months
Introduction conditions	Conditions under which animals are introduced into herd	no quarantine
		quarantine
Contact with other herds at watering points	Herd comes into contact with other herds at watering points	no
		yes
Contact with other herds during grazing	Herd comes into contact with other herds during grazing	no
		yes
Communal pen	Herds share a pen with other herds in the community	no
		yes
Antibiotics usage	Livestock farmer gives animals antibiotics	no
		yes

^1^could not cite any clinical signs.

^2^cited coughing & runny nose.

^3^cited coughing, difficulty breathing & runny nose.

The epidemiological unit was the herd; the variables were considered to be herd dependent and village independent.

### Data analyses

In the analyses, the 14 variables related to animal husbandry (Table 2) were the independent variables, and herd serological status (positive or negative) was the dependent variable.

The data were entered into Microsoft Excel files and then anonymized. Statistical analyses were performed using R (v. 4.2.0). To identify the variables influencing serological status, we examined the association of the variable values with herd seropositivity and seronegativity. For each variable, a Chi-squared test was performed to determine whether the observed proportions of herds testing seropositive or seronegative deviated significantly from the expected proportions across the variable values. Only variables with a *p*-value of < 0.2 were retained in the full logistic regression model to limit the risk of overfitting. This risk is inherent to automated variable selection methods based on AIC values^49^. Starting with this full model, we carried out automated model selection using the dredge function in the MuMIn package^50^. This function compares the AIC values of all possible submodels that can be built from the full model. Variable interactions were excluded to limit the risk of model overfitting. We examined the model with the lowest AIC value and any models that differed by less than 2.0 from the lowest AIC value. Among the latter, we defined the best-fit model as the one that included the most biologically relevant variables.

## Supplementary Information

Below is the link to the electronic supplementary material.

Supplementary Material 1: CBPP questionnaire, list of questions asked to breeders in order to collect the variables qualifying each herd.

Supplementary Material 2: Table S1. Results of Chi-squared analyses examining the association between the animal husbandry variables and herd seropositivity.

Supplementary Material 3: Table S2. Description of the six models including a panel of variables that were statistically associated with herd seropositivity, with the lowest AIC values and with the Odds Ratios related to the models having a Delta AIC lower than the retained model.

## Data Availability

The datasets used and analyzed in the study are available upon request by contacting the corresponding author.
