# Supplementary material for: Seroprevalence and potential risk factors of contagious bovine pleuropneumonia in the Huambo province of Angola
Source: Sci Rep. 2026 Apr 3;16:17652. doi: 10.1038/s41598-026-46690-9 (PMC13243479; doi:10.1038/s41598-026-46690-9)
Supplement: Supplementary file 1 — Supplementary Material 1: CBPP questionnaire, list of questions asked to breeders in order to collect the variables qualifying each herd. [file 41598_2026_46690_MOESM1_ESM.pdf]

First name/last name of respondent:

QUESTIONNAIRE

Location ____________________Date / __/______

Identifying information

Age:___________

Address:____

Respondent's occupation:____________________________________

Telephone number:____________________________________

- Have you received any agricultural training or training related to livestock farming? Yes □ No □
- Type of livestock farm: family □
- Total number of adult cattle (>12 months)__________

1. Presence of small ruminants in contact with cattle: Yes □ No □

If yes, how many:

2. Do you know what contagious bovine pleuropneumonia (CBPP) is? Yes □ No □

____

If yes, can you name a sign of the disease?_____________________________

____

If yes, can you say how the disease is transmitted?

____

3. Do you know if this disease has ever occurred in your herd? Yes □ No □ When:
4. Was vaccination performed during government campaigns? Yes □ No □

If not, where did you buy vaccines?__________________________

5. Date on which vaccination against CBPP was last carried out? ____

First name/last name of respondent:

6. Which group of animals is vaccinated?

   Adults □

   All □

1- Origin of animals.

7. Location where animals were purchased:

North □
South □
Southern Province (PS) □
North/PS □
South/PS □
North/South □

8. How often do you buy animals?

Once per year □

Twice per year □

Three times per year □

9. Date on which last animal was purchased?

< 6 months □ > 6 months □

10. What protocol do you use when introducing animals into the herd?

    Quarantine / other / no measures?

____

First name/last name of respondent:

Feed and water management:

11. When watering occurs in the environment (river, lake), do cattle come into contact with other animals? Yes □ No □
12. What type of feed do you give your livestock?

Hay, straw, silage □ Pasture □ Hay, straw, silage, and pasture □

13. Are pastures shared with other herds? Yes □ No □
14. The identity and length of the grazing period (month/year)? __
15. Communal or shared pen? Yes □ No □

Animal husbandry and veterinary assistance

16. Is there a veterinarian who works with the farm? Yes □ No □
    If yes, private or public?
    a. What is the frequency of visits per year?___________
17. When animals are sick, who makes the diagnosis?

Veterinarian □ Farmer □ Workers □

18. Who administers the treatment? Veterinarian □ Farmer □ Workers □
19. Do you use antibiotics? Yes □ No □
    a. Which antibiotics?____
    b. What is the average number of treatments per year for your herd?
20. Under what circumstances do you use antibiotics?

I decide it is needed □ Systematically when one of the cattle is sick □ Upon veterinary advice □

a. How do you store your antibiotics (describe your usage and storage practices)?

First name/last name of respondent:

Additional questions if cases of CBPP have previously occurred in the herd:

What did you do with the sick animals?
Left in contact with others ☐ Isolated and treated ☐ Sold ☐
Consumed☐ Other ☐...
If treatment occurred, what treatment did you use?
Modern ☐(antibiotics).. Traditional☐
Did a large number of deaths result following these respiratory illnesses?
Yes ☐ No☐
If so, approximately how many cases? ....
What did you do with the dead animals?
Discarded ☐ Landfill ☐ Incinerated ☐ Consumed ☐ Other ☐
....
Did you cut open the dead animals or did you have them cut open by a veterinary worker?
If so, what did you observe in the respiratory tract of the cadavers?
Unilateral lung involvement ☐ Presence of fibrin on the lung ☐
Fluid accumulation in pleura ☐ Presence of exudate in thoracic cavity ☐
Hepatization of the lung ☐ Sequestration ☐ Other
At what time of year do episodes of respiratory illness occur in the area?
J☐ F☐ M☐ A☐ M☐ J☐ Jy☐ A☐ S☐ O☐ N☐ D☐
Which animals on your livestock farm are most affected?
Less than 6 months ☐ Six months to one year ☐ More than a year ☐
Are there currently any animals with respiratory diseases in your herd?
Yes☐ No☐
