# Supplementary material for: Seroprevalence and potential risk factors of contagious bovine pleuropneumonia in the Huambo province of Angola
Source: Sci Rep. 2026 Apr 3;16:17652. doi: 10.1038/s41598-026-46690-9 (PMC13243479; doi:10.1038/s41598-026-46690-9)
Supplement: Supplementary file 2 — Supplementary Material 2: Table S1. Results of Chi-squared analyses examining the association between the animal husbandry variables and herd seropositivity. [file 41598_2026_46690_MOESM2_ESM.pdf]

Supplementary Table S1

Table S1: Results of Chi-squared analyses examining the association between the animal husbandry variables and herd seropositivity.

Variable	Variable value	No. herds	% Seropositive herds	p-value
Herd region	northern	75	57.3%	0.793
	southern	67	53.7%	
**Mixed herd**	no	121	51.2%	0.022
	yes	21	81.0%	
CBPP knowledge	good	35	57.1%	0.442
	poor	75	57.3%	
	none	27	44.4%	
	very good	5	80.0%	
Prior disease in herd	no	110	55.5%	1.000
	yes	32	56.2%	
Follows vaccination schedule	no	14	57.1%	1.000
	yes	128	55.5%	
**Presence of vaccinated animals**	adults only	118	51.0%	0.056
	all animals	24	75.0%	
**Purchase area**	northern Huambo	22	40.9%	0.036
	province south of Huambo	8	25.0%	
	province other than Huambo & northern Huambo	3	33.3%	
	province other than Huambo & southern Huambo	12	50.0%	
	southern Huambo	78	67.9%	
	northern & southern Huambo	19	42.1%	
Purchase frequency	once per year	40	52.5%	0.871
	twice per year	13	53.8%	
	three times per year	89	57.3%	
**Most recent animal purchase**	< 6 months	28	25.0%	0.001
	> 6 months	114	63.2%	
Introduction conditions	no quarantine	105	57.1%	0.676
	quarantine	37	51.4%	
Contact with other herds at watering points	no	46	50.0%	0.450
	yes	96	58.3%	
Contact with other herds during grazing	no	50	54.0%	0.911
	yes	92	56.5%	
Communal pen	no	131	56.5%	0.695
	yes	11	45.5%	
**Antibiotics usage**	no	51	68.6%	0.031
	yes	91	48.4%	
