# Supplementary material for: Seroprevalence and potential risk factors of contagious bovine pleuropneumonia in the Huambo province of Angola
Source: Sci Rep. 2026 Apr 3;16:17652. doi: 10.1038/s41598-026-46690-9 (PMC13243479; doi:10.1038/s41598-026-46690-9)
Supplement: Supplementary file 3 — Supplementary Material 3: Table S2. Description of the six models including a panel of variables that were statistically associated with herd seropositivity, with the lowest AIC values and with the Odds Ratios related to the models having a Delta AIC lower than the retained model. [file 41598_2026_46690_MOESM3_ESM.pdf]

Supplementary Table ST2

Description of the six models including a panel of variables that were statistically associated with herd seropositivity, with the lowest AIC values and with the Odds Ratios related to the models having a Delta AIC lower than the retained model.

Table 1: Description of the six models including a panel of variables that were statistically associated with herd seropositivity and with the lowest AIC values.

(Intercept)	Antibiotic use	Last purchase	Vaccine	mix	Purchase area	df	logLik	AICc	delta	weight
**-1.3391615**		**+**	**+**	**+**		**4**	**-78.07807**	**164.4814**	**0.0000000**	**0.33422593**
**-0.9292532**	**+**	**+**	**+**	**+**		**5**	**-77.47861**	**165.4490**	**0.9676626**	**0.20602261**
**-1.8395232**	**+**	**+**	**+**	**+**	**+**	**10**	**-72.12528**	**166.1309**	**1.6495577**	**0.14650190**
**-1.7332199**	**+**	**+**	**+**		**+**	**9**	**-73.43129**	**166.3880**	**1.9066488**	**0.12882989**
-1.2250881		+	+			3	-80.42126	167.0361	2.5547150	0.09317316
-2.1100512		+	+	+	+	9	-73.77622	167.0779	2.5965050	0.09124651

In bold, the models with the lowest AIC values within a meaningful ΔAIC ≤ 2 range. Highlighted in grey, prioritizing the model that included the most biologically relevant variables and the most comprehensive and informative.

Table 2 Odds ratios from the model of the relationship between the animal husbandry variables and herd-level CBPP seropositivity, with the lowest delta AIC.

factor	OR	p_val
(Intercept)	0.26 [0.09; 0.65]	0.006
mixO	3.49 [1.12; 13.47]	0.044
derni_acquis>6	4.37 [1.66; 12.77]	0.004
group._vaccinT	3.27 [1.16; 10.54]	0.033

Table 3 Odds ratios from the model of the relationship between the animal husbandry variables and herd-level CBPP seropositivity, with the second lowest delta AIC (*0.9676626)*.

factor	OR	p_val
(Intercept)	0.39 [0.11; 1.25]	0.126
mixO	3.35 [1.06; 13.06]	0.054
derni_acquis>6	3.97 [1.5; 11.66]	0.008
group._vaccinT	3.09 [1.08; 10.13]	0.045
antibioO	0.63 [0.27; 1.44]	0.275
